# LncRNA Nostrill promotes interferon-γ-stimulated gene transcription and facilitates intestinal epithelial cell-intrinsic anti-*Cryptosporidium* defense

**DOI:** 10.3389/fimmu.2024.1397117

**Published:** 2024-07-08

**Authors:** Zinat Sharmin, Kehua Jin, Ai-Yu Gong, Silu Deng, Chansorena Pok, Marion L. Graham, Shuhong Wang, Nicholas W. Mathy, Annemarie Shibata, Xian-Ming Chen

**Affiliations:** ^1^ Department of Microbial Pathogens and Immunity, Rush University Medical Center, Chicago, IL, United States; ^2^ Department of Biochemistry and Molecular Biology, School of Basic Medical Sciences, Hubei University of Science and Technology, Xianning, Hubei, China; ^3^ Department of Medical Microbiology and Immunology, Creighton University School of Medicine, Omaha, NE, United States; ^4^ Department of Biology, Creighton University College of Arts and Sciences, Creighton University, Omaha, NE, United States

**Keywords:** *cryptosporidium*, interferon, lncRNAs, Nostrill, cell-intrinsic defense, intestinal epithelium, transcription

## Abstract

Intestinal epithelial cells possess the requisite molecular machinery to initiate cell-intrinsic defensive responses against intracellular pathogens, including intracellular parasites. Interferons(IFNs) have been identified as cornerstones of epithelial cell-intrinsic defense against such pathogens in the gastrointestinal tract. Long non-coding RNAs (lncRNAs) are RNA transcripts (>200 nt) not translated into protein and represent a critical regulatory component of mucosal defense. We report here that lncRNA Nostrill facilitates IFN-γ-stimulated intestinal epithelial cell-intrinsic defense against infection by *Cryptosporidium*, an important opportunistic pathogen in AIDS patients and a common cause of diarrhea in young children. Nostrill promotes transcription of a panel of genes controlled by IFN-γ through facilitating Stat1 chromatin recruitment and thus, enhances expression of several genes associated with cell-intrinsic defense in intestinal epithelial cells in response to IFN-γ stimulation, including *Igtp*, *iNos*, and *Gadd45g*. Induction of Nostrill enhances IFN-γ-stimulated intestinal epithelial defense against *Cryptosporidium* infection, which is associated with an enhanced autophagy in intestinal epithelial cells. Our findings reveal that Nostrill enhances the transcription of a set of genes regulated by IFN-γ in intestinal epithelial cells. Moreover, induction of Nostrill facilitates the IFN-γ-mediated epithelial cell-intrinsic defense against cryptosporidial infections.

## Introduction

Cell-intrinsic immunity denotes the capacity of a host cell to eliminate an invasive/intracellular infectious agent at the cellular level which is a first line of host defense against intracellular pathogens including intracellular parasites (1). Intestinal epithelial cells are equipped with the necessary molecular machinery to mount cell-intrinsic defensive responses by themselves. Activation of pattern recognition receptors in intestinal epithelial cells leads to the upregulation of antimicrobial factors, secretion of cytokines and chemokines, and priming of immune cells for direct antimicrobial action or for guiding adaptive immune responses. On the other hand, intestinal epithelial cells serve as targets of mucosal immune mediators released from immune cells residing in the mucosa (2). IFNs have been identified as key elements of epithelial cell-intrinsic defense against intracellular pathogens in both mice and humans (3, 4).

The IFN family can typically be categorized into three main types: type I (e.g., IFN-α and IFN-β), type II (IFN-γ), and type III (IFN-λ family) (5). Type I IFNs bind to the conserved receptor IFNAR1/2 to induce transcription of type I IFN-stimulated genes (6). Although type III IFNs bind to the IFNLR1/IL-10RB receptor, they induce highly similar transcriptional responses as type I IFNs (5). IFN-γ binds to the heterodimeric receptor of IFNGR1 and IFNGR2, initiating the transcription of IFN-γ-stimulated genes (ISGs) (5). Canonical IFN-γ signaling employs the JAK/STAT pathway, leading to the activation of STAT1 and subsequent formation of active STAT1 homodimers. These homodimers then bind to gamma-activated sites (GASs) located in the promoters or enhancers of ISGs (6, 7). While the activation of various types of IFN signaling pathways is not mutually exclusive, mounting evidence suggests that IFN-γ plays a crucial role in anti-bacterial and anti-parasite immunity, whereas type I and type III IFNs are primarily associated with anti-viral immunity (3–6). Nonetheless, dysregulation of IFN signaling pathways can have detrimental effects on host defense mechanisms (7, 8). Therefore, activation of the IFN signal pathway is finely controlled and crosstalk between various types of IFN signaling has been demonstrated (6–9). Intestinal epithelial cells have the capacity to generate various type I and III IFNs, exerting autocrine effects (9). Although intestinal epithelial cells themselves do not produce IFN-γ, it is well established that IFN-γ produced from immune cells residing at the intestinal epithelium is essential to intestinal epithelial antimicrobial defense (10). IFN-γ triggers a broad spectrum of cell intrinsic responses aimed at combating intracellular pathogens through mechanisms like nutrient deprivation and the production of potent defense molecules, including reactive oxygen and nitrogen species, immunity-related GTPases (IRGs), and guanylate-binding proteins (GBPs) (3, 4, 8–10). However, numerous intracellular pathogens have developed sophisticated strategies to escape the immune response (11, 12). The specific regulatory components that govern IFN-γ-mediated defense by intestinal epithelial cells against diverse pathogens remain elusive.

Enteric infections caused by a growing spectrum of bacterial, parasitic, and viral pathogens exert significant impacts on intestinal absorption, nutritional status, childhood development, and contribute substantially to global mortality rates (13). *Cryptosporidium*, a coccidian parasite and an NIAID Category B priority pathogen, infects the intestinal epithelium and is a leading cause of infectious diarrhea and diarrheal-related death in children worldwide (14–16). Moreover, this infection can precipitate life-threatening diarrheal illnesses in individuals with AIDS (16). The majority of human *Cryptosporidium* infections are caused by two species: *C. parvum* and *C. hominis* (14), through ingesting *Cryptosporidium* oocysts. After excystation in the gastrointestinal tract to release infective sporozoites, each sporozoite then attaches to the apical membrane of intestinal epithelial cells and forms a parasitophorous vacuole, preventing further infection of other cell types (14). Consequently, intestinal epithelial cells serve as the frontline defense, with epithelial cell-intrinsic defense mechanisms playing a pivotal role in initiating, modulating, and resolving both innate and adaptive immune responses to *Cryptosporidium* infection (17, 18). IFN-γ is a key regulator of cell-intrinsic defense to *Cryptosporidium* infection (17, 18).

Long non-coding RNAs (lncRNAs), RNA transcripts exceeding 200 nucleotides in length, are notable for their non-protein-coding nature, yet many exhibit functional roles (19, 20). Accumulating evidence underscores the critical regulatory contribution of lncRNAs to mucosal defense mechanisms (21, 22). In our previous studies, we identified a lncRNA originating from the *2500002B13Rik* gene, which we termed Nostrill (iNOS transcriptional regulatory intergenic lncRNA locus) (23). We found that Nostrill may facilitate intestinal epithelial defense against *C. parvum* through regulation of the interferon response, albeit the underlying mechanisms remained elusive (24). In this study, we explore the role for Nostrill in IFN-γ-stimulated gene transcription and antimicrobial defense. Our findings indicate that Nostrill promotes the transcription of a subset of genes stimulated by IFN-γ and that the induction of Nostrill enhances the IFN-γ-mediated intestinal epithelial cell-intrinsic defense against *Cryptosporidium* infection.

## Results

### Nostrill knockdown alters gene expression profiles in intestinal epithelium following *Cryptosporidium* infection

**Figure 1:**
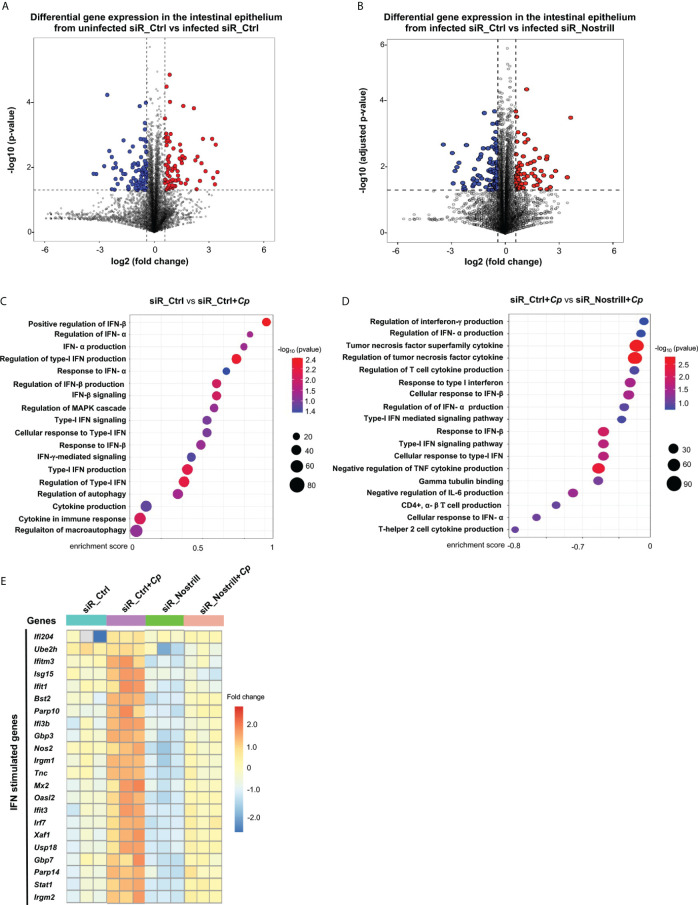
Transcriptomic responses in *Cryptosporidium*-infected IEC4.1 cells with Nostrill knockdown. **(A, B)** Volcano plots illustrating the differential gene expression between two groups. Statistical analysis utilized two-tailed Wald tests. The dashed line represents a false discovery rate cutoff of 0.05. Data were derived from three biological replicates for each group (p < 0.05 with a fold change > 1). Differentially expressed genes between infected siR_Ctrl (24h post-infection) versus uninfected siR_Ctrl (non-specific control siRNA) **(A)** and between infected siR_Nostrill versus infected siR_Ctrl (siRNA to Nostrill) **(B)** are shown. **(C, D)** Gene Set Enrichment Analysis (GSEA) depicting immune defense-related pathways in IEC4.1 cells from infected siR_Ctrl (24h post-infection) compared to uninfected siR_Ctrl **(C)** and in infected siR_Ctrl compared to infected siR_Nostrill (24h post-infection) **(D)**. p-values were calculated using the Kolmogorov–Smirnov test and adjusted via the Benjamini–Hochberg method. Enrichment scores and -log10 p-values for signaling pathways are presented. Red dots indicate smaller p-values, while blue dots indicate larger p-values. **(E)** Heatmap displaying representative transcriptional expression patterns for interferon-stimulated genes (ISGs) across different groups.

Several signaling pathways associated with cell-intrinsic defense are activated in the intestinal epithelium against *Cryptosporidium* following its infection, including the TLR/NF-кB signaling and IFN signaling (17, 18). Whereas signaling of all IFN types are activated in the intestine during *in vivo Cryptosporidium* infection, only IFN-I and IFN-III are activated *in vitro* infection models using intestinal epithelial cells because these cells in culture themselves cannot produce IFN-γ (10, 17, 18). In our previous studies, we demonstrated that knockdown of the lncRNA Nostrill using siRNA leads to a higher parasite burden of *Cryptosporidium* in intestinal epithelial cells (24). This suggested that Nostrill might play a role in the cell-intrinsic anti-parasitic defense. Expanding on this, we carried out comprehensive study by examining the transcriptome of cultured mouse intestinal epithelial IEC4.1 cells cells following *C. parvum* infection at a genome-wide level. These cells were subjected to treatment with a pool of siRNAs to knockdown Nostrill (referred as siR_Nostrill), followed by exposure to *C. parvum* infection for 24h. Consistent with our previous observations (25), siRNA knockdown of Nostrill in IEC4.1 cells increased the parasite infection burden (**Supplementary Figure 1**). The infection also triggered substantial changes in the gene expression patterns in IEC4.1 cells. Specifically, while comparing the gene expression between cells treated with siR_Ctrl (scramble siRNA control) with or without *C. parvum* infection, we identified a significant alteration in the expression levels of 11,761 genes. This shift encompassed both downregulation (5,590 genes) and upregulation (6,171 genes), demonstrating statistical significance at p<0.05 and a fold change >1 (**Figure 1A**). Intriguingly, the extent of gene alterations escalated further in infected cells subjected to siR_Nostrill treatment, revealing a higher count of changes. This encompassed both downregulated (5,791 genes) and upregulated (7,174 genes) entities, all adhering to the same rigorous statistical criteria (**Figure 1B**).

Next, through Gene Set Enrichment Analysis (GSEA), we investigated the gene expression profiles in IEC4.1 cells treated with the siR_Ctrl upon infection. In consistent with results from previous studies (17, 18), our analysis revealed activation of several innate defense pathways at the cellular level, such as IFN-mediated signaling and other cytokine-mediated signaling, in the infected cells (**Figure 1C**). Nonetheless, it was observed that pathways related to cellular defense appeared to be suppressed in cells treated with siR_Nostrill following infection, compared with infected cells treated with siR_Ctrl (**Figure 1D**). Genes whose expression levels were significantly altered and differentially expressed between siR_Ctrl-treated IEC4.1 and siR_Nostrill-treated IEC4.1 cells following infection were listed in **Supplementary Table 1**. It’s noteworthy that many of the well-known ISGs exhibited significant changes unique to each sample type. To visualize these changes, we employed unsupervised hierarchical clustering analysis, which generated a heat map illustrating the expression patterns of these differentially expressed immune defense genes, including many ISGs (**Figure 1E**). Intriguingly, genes such as *Ifi204, Irgm1/2, Nos2, Gbp3/7, Usp18, Irf7, Stat1*, and *Isg15*, which were upregulated in response to infection in cells treated with siR_Ctrl, showed decreased upregulation in cells treated with siR_Nostrill following infection. These findings highlight distinct gene expression profiles between infected siR_Ctrl-treated and siR_Nostrill-treated IEC4.1 cells, indicating a compromised cell-intrinsic defense response in cells treated with siR_Nostrill.

### Nostrill modulates the gene expression pattern of intestinal epithelial cells following IFN-γ stimulation

**Figure 2:**
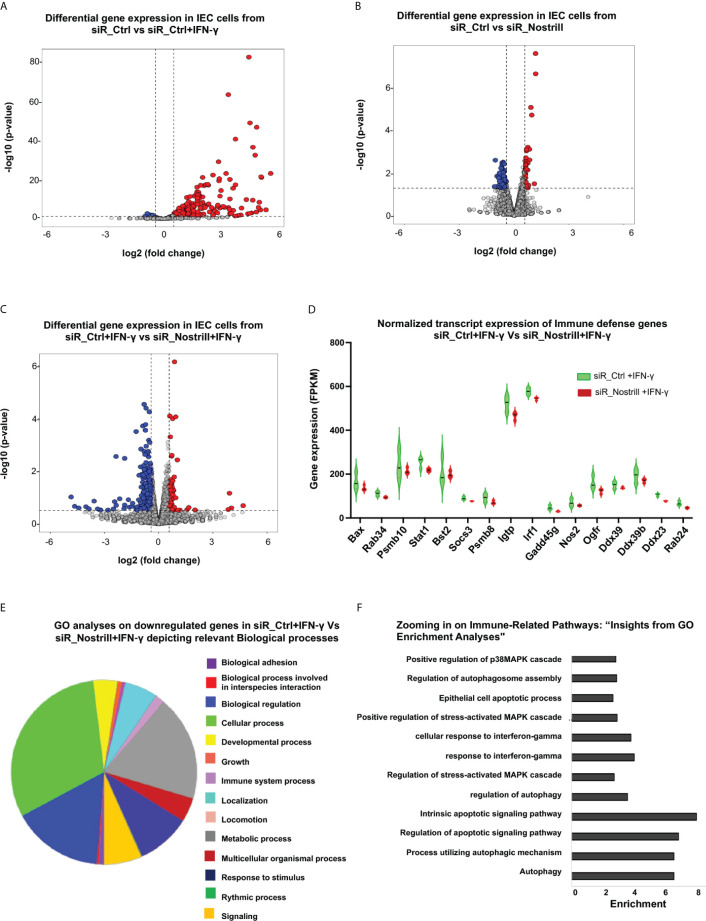
Impact of Nostrill on gene expression in intestinal epithelial cells upon IFN-γ stimulation. **(A-C)** Volcano plots illustrating the differential gene expression between two groups. Statistical analysis utilized two-tailed Wald tests. The dashed line represents a false discovery rate cutoff of 0.05. Data were derived from three biological replicates for each group (p < 0.05 with a fold change > 0.5). Differentially expressed genes between cells treated with siR_Ctrl + IFN‐γ (4 h post-treatment at a dose of 1 ng/ml) versus with siR_Ctrl **(A, B)** cells treated with siR_Nostrill versus siR_Ctrl (without IFN-γ treatment), and cells treated with siR_Nostrill + IFN‐γ (4 h post-treatment at a dose of 1 ng/mL) versus with siR_Ctrl + IFN‐γ **(C)** are shown. **(D)** Violin plot depicting the comparison of normalized transcript expression of host defense genes between siR_Ctrl- or siR_Nostrill-treated cells in response to IFN‐γ stimulation (4 h post-treatment at a dose of 1 ng/ml). **(E)** Gene ontology analyses using PANTHER GO-Slim tool revealed biological processes associated with downregulated genes in siR_Nostrill + IFN‐γ group when compared with that of siR_Ctrl + IFN‐γ group. **(F)** GO-KEGG analysis illustrating the enrichment of decreased Immune-related signaling pathways in response to siR_Nostrill + IFN‐γ treatment.

We then asked whether Nostrill induction acts as a host defense response to *Cryptosporidium* infection and can modulate IFN-mediated cell-intrinsic anti-parasitic defense in intestinal epithelial cells. Whereas signaling of all IFN types are activated in the intestine during *in vivo Cryptosporidium* infection, only IFN-I and IFN-III are activated *in vitro* infection models using intestinal epithelial cells because these cells in culture themselves cannot produce IFN-γ (10). Taken advantage on the absence of IFN-γ in the *in vitro* infection models, we investigated the impact of Nostrill induction on cell-intrinsic anti-parasitic defense in response to exogenous IFN-γ using our *in vitro* IEC4.1 cell infection model. We first performed a genome-wide transcriptome analysis of cultured IEC4.1 cells treated with the siR_Ctrl or siR_Nostrill in response to IFN-γ. Upon comparing the gene expression patterns between the siR_Ctrl-treated cells with and without IFN-γ stimulation, we have identified many genes that displayed significant differential expression (p<0.05, fold change > 0.5) (**Figure 2A**). Most of these genes are upregulated, confirming the impact of IFN-γ treatment on the cells in consistent with previous studies (26–28). When comparing the basal gene expression levels between the siR_Ctrl- and siR_Nostrill-treated cells, without IFN-γ treatment, we observed 186 genes that exhibited differential expression (**Figure 2B**). Nevertheless, when comparing expression levels of genes in the siR_Ctrl + IFN-γ-treated cells versus siR_Nostrill + IFN-γ-treated cells, a total of 1,559 genes showed significant differential expression, indicating a notable impact of Nostrill on the gene expression induced by IFN-γ treatment (**Figure 2C**). Out of these genes, 883 were genes whose expression levels were significantly lower in the siR_Nostrill + IFN-γ-treated cells than that in the siR_Ctrl + IFN-γ-treated cells (**Figure 2C**). The normalized transcript expression levels of selected genes out of these 883 genes were shown in **Figure 2D**, including *Igtp*, *iNos*, and *Gadd45g*. The complete dataset, containing a comprehensive list of genes showing differential expression between these groups, is available in the **Supplementary Table 2**.

We then performed gene ontology (GO) analysis to understand the functional enrichment and biological pathways associated with these differentially expressed genes associated with Nostrill in IFN-γ-treated cells. The PANTHER GO-slim tool revealed that signaling pathways associated with cellular process, biological regulation, and immune system process were generally suppressed in siR_Nostrill-treated IEC4.1 cells upon IFN-γ stimulation, compared with that in siR_Ctrl- and IFN-γ-treated cells (**Figure 2E**). To get a deeper understanding of these immune system processes, we integrated GO and Kyoto Encyclopedia of Genes and Genomes (KEGG) databases to perform functional analysis. The GO KEGG analysis revealed various immune system processes that were downregulated in siR_Nostrill-treated cells in response to IFN-γ, including IFN-γ-mediated cellular responses, regulation of MAPK cascade and autophagy mediators (**Figure 2F**). Expression levels of selected genes associated with cell-intrinsic defense were further validated at the RNA level in cells treated with siR_Nostrill or cells overexpressing Nostrill, using quantitative real-time reverse transcription PCR (qRT-PCR) (**Supplementary Figure 1**).

### Nostrill facilitates chromatin recruitment of Stat1 to promote IFN-γ-mediated gene transcription in intestinal epithelial cells

**Figure 3:**
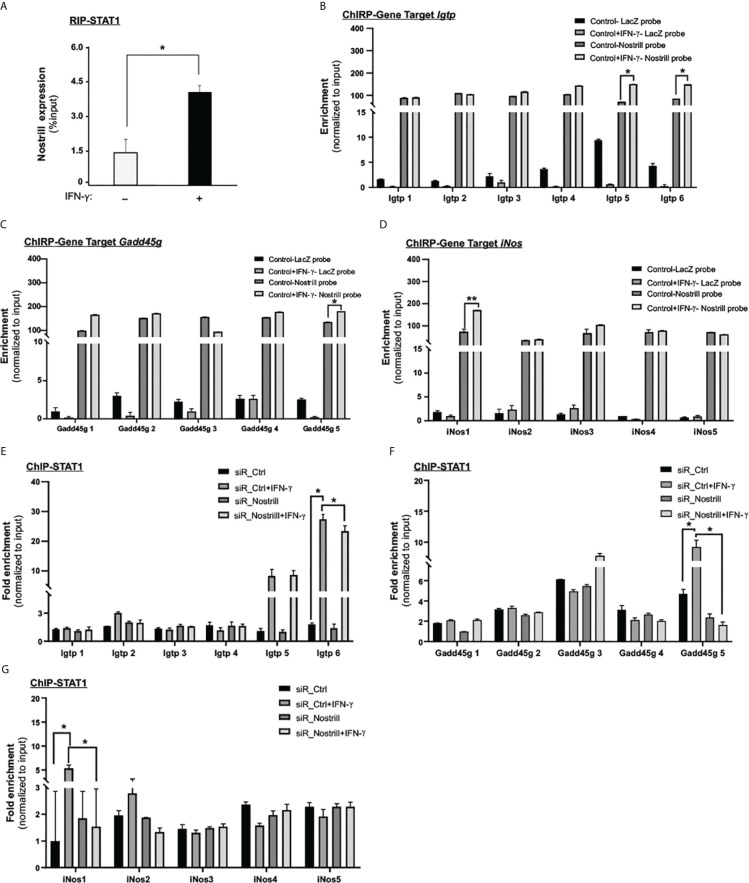
Molecular insights into Nostrill and STAT1 interaction in regulating ISG transcription. **(A)** Evidence of physical interaction between Nostrill and p65 in intestinal epithelial cells. IEC4.1 cells were exposed to a 5 ng/mL dose of IFN‐γ for 2h, followed by RNA immunoprecipitation (RIP) analysis utilizing an anti-Stat1 antibody. The presence of Nostrill was quantified through qRT-PCR and normalized against the percent input. Significance (*p < 0.05) was determined when compared to the control between the indicated groups. **(B–D)** Recruitment of Nostrill to ISG promoter sites following IFN‐γ treatment. IEC4.1 cells were exposed to a 5 ng/mL dose of IFN‐γ for 2h, and subsequent ChIRP analysis was carried out using pool of probes specific to Nostrill, along with designed PCR primer sets targeting Stat1 binding sites of *Igtp, Gadd45g*, and *iNos*. A pool of Lacz probes was used as negative control. Significance levels (*p < 0.05) were assessed in comparison to the control. **(E–G)** Influence of Nostrill siRNA knockdown on Stat1 recruitment to *Igtp, Gadd45g*, and *iNos* promoter sites upon IFN‐γ stimulation. IEC4.1 cells were transfected with siR_Nostrill or siR_Ctrl for 24h, then exposed to IFN‐γ for 2h, followed by ChIP analysis using anti-p65 antibody and designed PCR primer sets. Statistical significance (*p < 0.05) was determined in comparison to siR_Ctrl. Data are presented as means ± SEM with “n” of 3.

The canonical IFN-γ signaling utilizes the JAK/STAT pathway to activate Stat1, resulting in the formation of active Stat1 homodimers (5). This complex then binds to gamma-activated sites in the promoters/enhancers of ISGs (5). To determine whether Nostrill and the Stat1 transcription factor have a direct physical relationship with one another, we performed formaldehyde crosslinking RNA immunoprecipitation (RIP) analysis. We observed that the anti-Stat1 immunoprecipitates from IFN-γ-stimulated cells contained a significant amount of Nostrill. In IFN-γ-stimulated cells, immunoprecipitation of Stat1 indicated a 1.8-fold increase in Nostrill enrichment as compared to the untreated control cells (**Figure 3A**).

To determine if Nostrill binds to specific genomic sites in the promoters of ISGs, potentially facilitating the binding of Stat1, we employed the Chromatin Isolation by RNA Purification (ChIRP) technique. We used biotinylated probes specific to Nostrill to isolate chromatin fragments and then measured the enrichment of Nostrill to the promoter regions of selected ISGs, such as, *Igtp*, *iNos*, and *Gadd45g*. A pool of eight biotinylated tiling oligonucleotide probes unique to Nostrill were utilized to isolate the associated chromatin fragments and a pool of six LacZ probes were used as control. Following the Nostrill pull-down, we conducted qRT-PCR assays employing primers designed to target the Stat1 binding sites within the respective gene promoters. The findings revealed enhanced associations of Nostrill with the promoter regions of these genes in response to IFN-γ stimulation (**Figures 3B–D**). Notably, there was an amplified interaction between Nostrill and the *Igtp* promoter regions 5 and 6 following IFN-γ stimulation (**Figure 3B**). In the case of the *Gadd45g* promoter site 5, a noticeable increase in Nostrill abundance was observed (**Figure 3C**). Likewise, the *iNos* promoter region 1 exhibited an increased enrichment of Nostrill upon IFN-γ stimulation (**Figure 3D**).

To further investigate the potential involvement of Nostrill in docking Stat1 at the promoter regions of three genes: *Igtp*, *iNos*, and *Gadd45g*, we employed chromatin immunoprecipitation (ChIP) to assess Stat1 recruitment in IEC4.1 cells treated with siR_Nostrill (**Figures 3E, F**). The results showed that in siR_Ctrl-transfected cells stimulated with IFN-γ, there was a significant increase of Stat1 enrichment at the site 5 and site 6 regions of the *Igtp* promoter compared to the non-IFN-γ-treated control (p = 0.05) (**Figure 3E**). This suggests that Stat1 plays a role in the transcriptional regulation of *Igtp* in response to IFN-γ. Furthermore, when Nostrill expression was silenced in IEC4.1 cells and then exposed to IFN-γ stimulation, the enrichment of Stat1 at the site 6 region of the *Igtp* promoter was significantly reduced (p = 0.02). Of note, Nostrill was also recruited to the same site 6 region in the IFN-γ-treated IEC4.1 cells (**Figure 3B**). This indicates that Nostrill is involved in the docking or recruitment of Stat1 to the *Igtp* transcriptional region, potentially acting as a co-regulator. Similar observations were made for the *Gadd45g* and *iNos* promoter regions. Following IFN-γ stimulation, enrichment of Stat1 at the site 5 region of the *Gadd45g* promoter showed a significant increase (**Figure 3F**), while at the site 1 region of the *iNos* promoter, it increased significantly in the siR_Ctrl-transfected IEC4.1 cells (**Figure 3G**). However, silencing of Nostrill in cells significantly reduced the enrichment of Stat1 at these corresponding regions of the *Gadd45g* and *iNos* promoters (**Figures 3F, G**). Recruitment of Nostrill was observed to the same regions of the *Gadd45g* and *iNos* promoters in IFN-γ-treated IEC4.1 cells (**Figures 3C, D**). These findings suggest that Nostrill interacts with Stat1 and may function as a co-regulator for the chromatin enrichment of Stat1 to promote IFN-γ-stimulated gene transcription in intestinal epithelial cells.

### Nostrill promotes IFN-γ-mediated intestinal epithelial cell-intrinsic anti-*Cryptosporidium* defense

**Figure 4:**
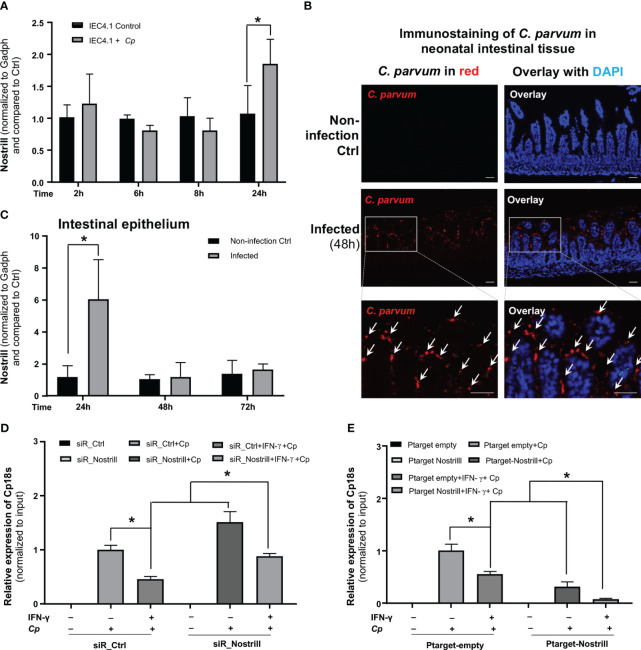
Impact of Nostrill on *C. parvum* infection burden in intestinal epithelial cells in response to IFN-γ. **(A)** Upregulation of Nostrill in cultured intestinal epithelial cells following exposure to *C. parvum* infection. IEC4.1 cells were infected with *C. parvum* for 2-24h and the level of Nostrill was measured via qRT-PCR. **(B)** *C. parvum* infection of the intestinal epithelium in neonatal mice. Neonatal mice, aged 5 days, were orally exposed to *C. parvum*, while control mice received PBS. *C. parvum* infection was confirmed by immunostaining of the parasite in the infected intestinal epithelium (48h post-infection). *C. parvum* was stained in red with the counterstaining of cell nuclei with DAPI in blue. Representative images are shown. Bar = 50 µm. **(C)** Nostrill induction in the intestinal epithelium from infected neonatal mice in response to *C. parvum* infection. Intestinal epithelium samples were collected at 24, 48, and 72h post-infection, and Nostrill expression was quantified using qRT-PCR. **(D, E)** Impact of Nostrill on *C. parvum* infection burden, **(D)** Effect of Nostrill knockdown on *C. parvum* infection burden in a cell culture. IEC4.1 cells were transfected with either siR_Ctrl or siR_Nostrill. Subsequently, they were exposed to IFN-γ 10 ng/ml for 24h and then infected with *C. parvum* for 8h. Quantification of *C. parvum* infection was performed using qRT-PCR. **(E)** The impact of Nostrill overexpression on *C. parvum* infection *in vitro*. IEC4.1 cells were transfected with a Nostrill overexpression plasmid (PTarget-Nostrill) or an empty vector control (PTarget-Empty). After 24h of treatment with IFN-γ 10 ng/ml, the cells were infected with *C. parvum* for 8h, and the extent of infection was quantified via qRT-PCR. Statistical significance was calculated using *t*-test. Significance levels (*p < 0.05). Data are presented as means ± SEM with “n” of 3.

Our lab has previously demonstrated the induction of Nostrill in intestinal epithelial cells in response to *C. parvum* infection (24). To this end, IEC4.1 cells were infected with *C. parvum* for up to 24h. At 24h following infection, the Nostrill’s induction was apparent, demonstrating a 72% increase in its expression (**Figure 4A**). To investigate the expression profile of Nostrill in a relevant *in vivo* model and gain insights into its potential role in the context of intestinal epithelium, neonatal mice (5 days old) were infected with *C. parvum* via oral gavage. Infection of the small intestinal epithelium by *C. parvum* was evident by immunostaining (**Figure 4B**). Consistent with previous reports (24), the expression of Nostrill was significantly increased at 24h after the infection in the intestinal epithelium (**Figure 4C**).

Given the significant impact of Nostrill on IFN-γ-mediated gene transcription in intestinal epithelial cells, we sought to ask whether Nostrill induction may influence IFN-γ-mediated intestinal epithelial cell-intrinsic defense against *C. parvum* infection. To investigate this, we initially primed siR_Ctrl- and siR_Nostrill-treated IEC4.1 cells with IFN-γ and subsequently infected them with *C. parvum*, followed by an assessment of the resulting infection burden. Consistent with previous reports (25, 29), we detected a decreased infection burden in siR_Ctrl-treated IEC4.1 cells primed with IFN-γ (**Figure 4D**). Intriguingly, knockdown of Nostrill with the siRNA in IEC4.1 cells resulted in a significant suppression of IFN-γ-mediated epithelial defense, reflected by a higher infection burden in siR_Nostrill-treated and IFN-γ-primed cells compared with that in IFN-γ-primed cells treated with the siR_Ctrl (**Figure 4D**). Complementarily, overexpression of Nostrill resulted in enhanced IFN-γ-mediated inhibition of infection burden in IEC4.1 cells (**Figure 4E**). These data suggest that Nostrill promotes IFN-γ-mediated epithelial cell-intrinsic anti-*Cryptosporidium* defense.

### Nostrill exerts anti-*Cryptosporidium* defense through IFN-γ-induced autophagy

**Figure 5:**
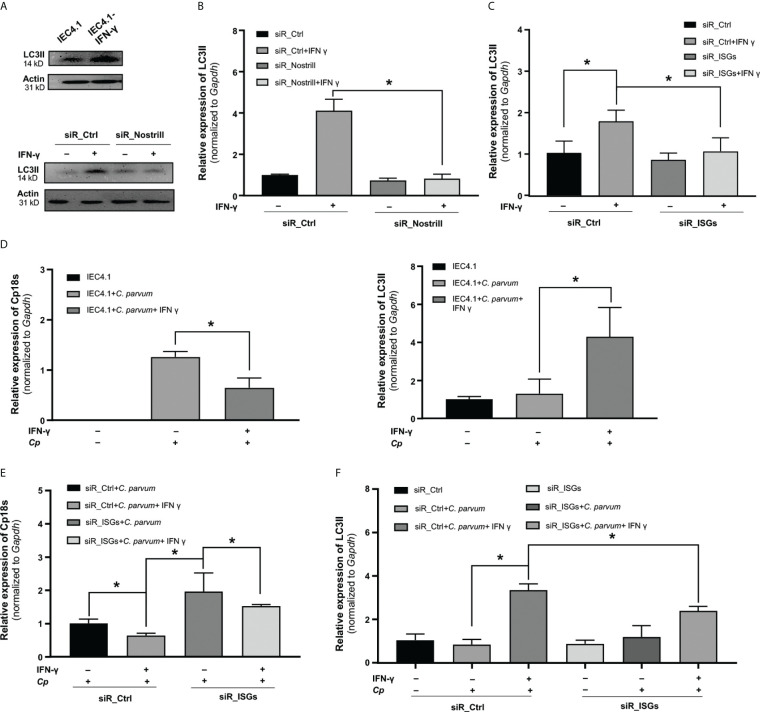
Evidence for Nostrill-mediated enhancement of anti-*Cryptosporidium* defense via IFN-γ-induced autophagy in intestinal epithelial cells. **(A, B)** Assessment of autophagy in Nostrill knockdown cells. **(A)** IEC4.1 cells were exposed to IFN-γ for 24h, followed by Western blot analysis to evaluate the autophagy marker LC3II (14kDa), with beta-actin (31kD) serving as a reference protein. A similar analysis was conducted on Nostrill-knocked down cells exposed to IFN-γ. **(B)** Quantification of LC3II levels in Nostrill knockdown cells was performed via qPCR. **(C)** Impact of Nostrill associated genes on autophagy. A combination of siRNAs was used to knockdown *Igtp, iNos*, and *Gadd45g (*referred to as siR_ISGs) in IEC4.1 cells, followed by treatment with 10 ng/ml of IFN-γ for 24h. The level of LC3II marker was quantified through qRT-PCR. **(D-F)** Impact of Nostrill associated ISGs in defending *C. parvum* through autophagy. **(D)** IEC cells were treated with 10 ng/ml of IFN γ for 24h and then infected with *C. parvum* for 8h. Level of *Cp18s* and *LC3II* were measured by qRT-PCR. **(E, F)** IEC4.1 cells were treated with siR_ISGs (260 nM), which consisted of a pool of siRNAs targeting *Igtp* (80 nM), *iNos* (60 nM), and *Gadd45g* (120 nM), or a scrambled siR_Ctrl at 260 nM. Subsequently, cells were treated with 10 ng/ml of IFN-γ for 24h and then infected with *C. parvum* for 8h. **(E)** Cp18s, representative of *C. parvum* burden, and **(F)** LC3II levels as an indicator of autophagy were quantified using qRT-PCR. Statistical significance was calculated using *t*-test. Significance levels (*p < 0.05). Data are presented as means ± SEM with “n” of 3.

Autophagy has been recognized as one of the immune responses elicited by IFN-γ against intracellular pathogens (30). Several genes, whose expression in response to IFN-γ in intestinal epithelial cells was associated with Nostrill, including *Igtp*, *iNos*, and *Gadd45g*, have been implicated in the regulation of cellular autophagy (30, 31). To examine the role of Nostrill in IFN-γ-induced autophagy, we first assessed the autophagy levels in IEC4.1 cells following exposure to IFN-γ at a dose of 10 ng/mL for 24h. We quantified autophagosome formation using LC3-II release as the marker as previously reported (32, 33). Our results clearly demonstrated the induction of autophagy in IEC4.1 cells following IFN-γ stimulation, as corroborated by both Western blot and qRT-PCR analyses of LC3-II release (**Figures 5A, B**, **Supplementary Figure 2**). Subsequently, we sought to determine the influence of Nostrill on this process. To do so, we utilized siR_Nostrill to knockdown its expression in the cells before exposure to IFN-γ. Our findings revealed a significant inhibition of IFN-γ-induced autophagy in cells treated with siR_Nostrill (**Figures 5A, B**).

To further investigate the contribution of Nostrill-mediated genes, including *Igtp*, *iNos*, and *Gadd45g*, to IFN-γ-induced autophagy, we employed a combination of siRNAs (siR_ISGs) to silence their expression in IEC4.1 cells (**Supplementary Figure 3**). We then examined the impact of siR_ISG knockdown on IFN-γ-induced autophagy. Silencing *Igtp, iNos*, and *Gadd45g* with the siR_ISGs led to a significant inhibition of IFN-γ-induced autophagy (**Figure 5C**).

The subsequent question we aimed to address was whether these Nostrill-associated ISGs within the IFN-γ pathway play a role in combating *Cryptosporidium* infection through autophagy. To investigate this, we first assessed the expression of LC3II in IEC4.1 cells treated with IFN-γ and subsequently infected with *C. parvum*. As shown in **Figure 5D**, IFN-γ treatment led to a reduction in *C. parvum* burden in the cells. Meanwhile, there was a significant increase in the LC3II levels in the IFN-γ-treated cells following *C. parvum* infection (**Figure 5D**). This suggests that IFN-γ may reduce *C. parvum* burden in the cells through induction of autophagy. To further substantiate the significance of IFN-γ-induced autophagy in the defense against *Cryptosporidium*, we assessed both the *Cryptosporidium* infection burden and the LC3II levels in cells with or without treatment with the siR_ISGs. IEC4.1 cells were first treated with siR_Ctrl or siR_ISGs for 24h, pre-treated with IFN-γ for additional 24h, and subsequently infected with *Cryptosporidium* for 24h. Similar to our observations in Nostrill knockdown cells, the knockdown of selected ISGs in IEC4.1 cells resulted in a significant suppression of IFN-γ-mediated epithelial defense, as evidenced by a higher infection burden in the siR_ISGs-treated and IFN-γ primed cells compared to those treated with the siR_Ctrl (**Figure 5E**). Furthermore, we observed a reduction in LC3II levels in cells transfected with siR_ISGs, specifically when treated with IFN-γ and subsequently infected with *C. parvum* (**Figure 5F**). Taken together, the above data suggest that Nostrill facilitates the transcription of *Igtp, Gadd45g*, and *iNos* induced by IFN-γ, which in turn positively regulates autophagy, ultimately contributing to the epithelial cell-intrinsic defense against *Cryptosporidium* infection.

## Discussion

Intestinal epithelial cell-intrinsic immunity provides the first line of host defense against intracellular pathogens in the intestine (2). This defense mechanism operates through layered innate immune signaling networks within intestinal epithelial cells, which detect microbial pathogens and trigger downstream mechanisms for pathogen eradication. These mechanisms include the production of antimicrobial proteins, activation of specialized degradative compartments, and initiation of programmed host cell death. IFNs stand as one of the most potent signals derived from vertebrates, mobilizing antimicrobial effector functions against intracellular pathogens (2, 9) and serving as cornerstones of intestinal epithelial cell-intrinsic defense. Emerging evidence highlights the critical role of long lncRNAs as regulatory components in gastrointestinal defense against microbial infection (22, 24, 34, 35). In this study, we investigated the impact of Nostrill, a MyD88/NF-кB-associated lncRNA as we previously identified (23), on the regulation of IFN-γ-mediated antimicrobial defense in intestinal epithelial cells. Our findings demonstrate that Nostrill enhances the transcription of a set of genes regulated by IFN-γ in intestinal epithelial cells and induction of Nostrill facilitates IFN-γ-stimulated epithelial cell-intrinsic anti-cryptosporidial defense.

Several lncRNAs are induced in innate immune cells and are likely to play key roles in the regulation of innate defense. For instance, lncRNAs exhibit differential regulation in virus-infected cells (36) and in dendritic cells or macrophages upon stimulation by ligands for TLR4 and TLR2 (21). LncRNA-Cox2, among the most highly induced lncRNAs in macrophages, has been demonstrated to mediate both the activation and repression of distinct classes of immune genes (21, 37–39). Recent research has shown that various IFN responses are exquisitely regulated by lncRNAs and these IFN-related lncRNAs are also highly tissue- and cell-type-specific, rendering them as promising biomarkers or therapeutic candidates to modulate specific stages of the antiviral immune response with fewer adverse effects (40). LncRNAs are implicated in fundamental roles as inducers or repressors at nearly every stage of the IFN response. Our findings align with this notion, indicating that Nostrill can modulate gene transcription in intestinal epithelial cells in response to IFN-γ stimulation. Modes of action for lncRNAs encompass various mechanisms, such as regulation of gene transcription either in cis or in trans by recruiting proteins or molecular complexes to specific gene loci, acting as scaffolds for nuclear complexes, titrating RNA-binding proteins, and forming RNA-RNA pairs to induce posttranscriptional regulation (41). Our observations indicate that Nostrill facilitates the recruitment of Stat1 to the promoters of IFN-γ-stimulated genes. Similar mechanisms have been previously demonstrated with lncRNA XR_001779380 in regulating IFN-γ-stimulated gene transcription in intestinal epithelial cells (22). Intriguingly, Nostrill selectively promotes the transcription of a subset, rather than all, of IFN-γ-stimulated genes in IEC4.1 cells. In addition, not all the promoter regions with an increased enrihment of Stat1 are accompanied with an enhanced Nostrill recruitment. Obviously, besides Stat1 promoter recruitment, other determinants may be involved in Nostrill-mediated gene transcription in intestinal epithelial cells in response to IFN-γ stimulation. In our previous studies (24), we found that Nostrill can recruit IRF7 and NF-кB p65 to several defense gene loci in cells following cryptosporidial infection. It would be worthwhile to explore whether the recruitment of IRF7 and NF-кB p65 is also implicated in Nostrill-mediated gene transcription in intestinal epithelial cells in response to IFN-γ.

As a result, the induction of Nostrill may enhance IFN-γ-mediated cell-intrinsic defense against intracellular pathogens in intestinal epithelial cells by facilitating gene transcription in response to IFN-γ stimulation. Indeed, we observed a greater burden of *Cryptosporidium* infection (indicating diminished cellular defense) in intestinal epithelial cells treated with siRNA to knockdown Nostrill following IFN-γ stimulation, compared to control-siRNA-treated cells. Hence, the induction of Nostrill in intestinal epithelial cells following *Cryptosporidium* infection through activation of the MyD88/NF-кB pathway may prime host epithelial cells for a more efficient cell-intrinsic defense in response to IFN-γ released upon activation of immune cells residing at the epithelium. Induction of another lncRNA, XR_001779380, has been demonstrated to provide a similar promoting effect on cellular response to IFN-γ during *Cryptosporidium* infection in the intestine through promoting Swi/Snf-mediated gene transcription (22).

IFN-γ instigates a diverse array of cell-intrinsic responses aimed at inhibiting parasite growth through mechanisms such as nutrient deprivation and the production of potent defense molecules, including reactive oxygen and nitrogen species, IRGs, and GBPs (3, 4, 8–10). Notably, Nostrill can promote the transcription of many of these genes in intestinal epithelial cells following IFN-γ stimulation. Among them, certain genes such as *Igtp*, *iNos*, and *Gadd45g* are of particular interest due to their regulatory roles in autophagy. Their upregulation may contribute to enhanced cell-intrinsic defense in response to IFN-γ associated with Nostrill induction. Stimulation with IFN-γ induces autophagy in cultured intestinal epithelial cells, a process that is partially impeded by knockdown of *Nostrill*, *Igtp*, *iNos*, or *Gadd45g*. These findings suggest that Nostrill may augment IFN-γ-mediated cell-intrinsic defense against *Cryptosporidium* infection in intestinal epithelial cells by promoting the transcription of genes associated with autophagy. Autophagy serves as a critical defense mechanism against infections, facilitating the capture and elimination of intracellular parasites (42). Recent studies have reported the induction of autophagy in intestinal epithelial cells via mTOR inactivation during *Cryptosporidium* infection of Caco-2 cells (43). Diminished host cell autophagy can promote parasitic survival in host cells following *Cryptosporidium* infection (44, 45).

Given the fact that all three types of IFN signaling are activated in the intestinal epithelium following *Cryptosporidium* infection (25, 46–48), it would be intriguing to investigate whether Nostrill also regulates gene transcription in intestinal epithelial cells in response to stimulation by other type IFNs. While type II (IFN-γ) and III IFNs demonstrate a pronounced protective role (47–51), type I IFN signaling exhibits a pro-parasitic effect, as evidenced by the resistance of mice lacking Ifnar1 to *C. parvum* infection (25, 47). Furthermore, elucidating the role of Nostrill in regulating IFN-mediated intestinal epithelial anti-*Cryptosporidium* defense warrants further investigation using *in vivo* infection models employing conditional knockout strains of various IFN types.

In conclusion, our findings suggest that Nostrill may enhance intestinal epithelial defense against *Cryptosporidium* infection through its regulation of interferon responses. We investigated the role of Nostrill in IFN-γ-stimulated gene transcription and antimicrobial defense. Our results indicate that Nostrill promotes the transcription of a subset of genes controlled by IFN-γ, and its induction facilitates IFN-γ-mediated intestinal epithelial cell-intrinsic defense against *Cryptosporidium*. A deeper understanding of how lncRNAs like Nostrill modulate the transcription of host defense genes would offer new insights into mucosal immunity to *Cryptosporidium* and potentially other pathogens in general.

## Materials and methods

### Cell culture

Dr. Pingchang Yang of McMaster University in Hamilton, Canada, generously provided the newborn mouse intestinal epithelial cell line (IEC4.1) (52). Cultures of IEC4.1 cells were maintained in DMEM media with 10% fetal bovine serum (FBS; Gibco; Carlsbad, CA, USA). Streptomycin at 100 μg/ml and penicillin at 100 U/ml (both from Gibco) were added to the growth media. Tissue culture flasks were used to cultivate the cells in a 37°C, 5% CO_2_ incubator.

### *C. parvum* oocyst

*Cryptosporidium parvum* oocysts, sourced from the Iowa strain provided by Harley Moon at the National Animal Disease Center (Ames, IA), were purchased commercially from the Bunch Grass Farms in Deary, ID. To purify the oocysts, a modified ether extraction technique was employed. Subsequently, the oocysts were suspended in phosphate-buffered saline (PBS) and stored at a temperature of 4°C. For both *in vitro* and *in vivo* infection experiments, the oocysts were subjected to a treatment with 1% sodium hypochlorite while being preserved on ice for 20 minutes. After this treatment, the oocysts were washed three times using Dulbecco’s modified Eagle medium (DMEM) culture media.

### Infection models and assays

For *in vitro* infection studies using IEC4.1 cells, viable *C. parvum* oocysts were utilized. These oocysts were treated with 1% sodium hypochlorite and were added to a culture medium consisting of DMEM-F-12 supplemented with 100 U/ml penicillin and 100 μg/ml streptomycin. The infection was established by combining oocysts and host cells in a 1:1 ratio. The cell cultures were then incubated at 37°C for 4h to facilitate parasite attachment and invasion. After this, the cells were thoroughly washed with DMEM-F-12 medium three times to remove any free parasites. The cells were further cultured for varying time periods based on the experimental requirements. For the *in vivo* experiments, a neonatal murine model of intestinal cryptosporidiosis was employed (25). Neonates, aged 5 days after birth, were orally administered *C. parvum* oocysts (10^5 oocysts per mouse) through gavage to induce intestinal cryptosporidiosis. Control mice were given PBS via the same method. Following 24, 48, and 72h from the administration of *C. parvum* oocysts or PBS, the animals were sacrificed, and samples of ileum intestine tissues were collected. Epithelial tissues from the ileum were extracted from at least five animals within each experimental group. The assessment of *C. parvum* was done using qRT-PCR both *in vitro* and *in vivo* context.

### siRNA-mediated knockdown

To silence the Nostrill gene in murine cells, a pool of three small interfering RNA (siRNA) duplexes (siR_Nostrill) was synthesized using the WI siRNA Selection Program at the Massachusetts Institute of Technology. The specific siRNA sequences used were as follows: 25b_1: sense 5’ CCUGGGAAGAAGCAUUAAU 3’, 25b_2: sense 5’ CCACCAGGAACACACAAAU 3’, and 25b_3: sense 5’ GCCUAUAAGUCGUUCUAAU 3’. The IEC4.1 cells were cultured in 24-well plates at a density of 1.5 x 10^5 cells per milliliter. The next day, the cells were transfected with 120 nM of the siRNA pool (40 nM of each of the siRNA) using Lipofectamine RNAiMax from Life Technologies, Inc., following the manufacturer’s instructions. As a negative control, non-specific scrambled siRNA at a concentration of 120 nM was used as the control siRNA (siR_Ctrl). The transfection process was allowed to continue for 24h, after which the cells were treated with IFN-γ. RNA was then isolated from the cells using a previously described method.

*Igtp*, *iNos*, and *Gadd45g* were targeted for knockdown of ISGs in mouse IEC4.1 cells. The siRNA-mediated knockdown approach was employed to silence these genes. The siRNA to iNos was synthesized using the WI siRNA Selection Program at the Massachusetts Institute of Technology (MIT) and the sequence was sense 5’ GCCGCUCUAAUACUUAGCU 3’. For silencing Igtp and Gadd45g, siRNA duplexes were purchased from Santacruz Biotechnology with the catalogue numbers sc-41792 and sc-37419, respectively. The IEC4.1 cells were cultured in 24-well plates at a density of 1.5 x 10^5 cells/ml. After overnight incubation, the cells were transfected with a pool of the three sets of siRNAs to achieve knockdown of Igtp, Gadd45g, and iNos (as siR_ISGs). The concentrations of gene-specific siRNAs used for transfection were as follows: siR_Igtp at 80 nM, siR_Gadd45g at 120 nM, and siR_Nos at 60 nM. siR_Ctrl at a concentration of 260 nM was used for control. Lipofectamine RNAiMax from Life Technologies, Inc. was used as the transfection reagent, and the transfection process was carried out following the manufacturer’s instructions.

### RNA isolation

Total RNA from the cells was extracted using the TRIzol reagent from Invitrogen as per the manufacturer’s instructions. The concentration and quality of the RNA were assessed using a spectrophotometer from Beckman in Brea, CA. For subsequent steps, 500 ng of the RNA sample was utilized as a template for cDNA synthesis, which was performed using the M-MLV Reverse Transcriptase kit from Invitrogen, located in Carlsbad, CA. The reaction was carried out in a total volume of 20 µl.

### qRT-PCR

The qRT-PCR was conducted using the Invitrogen™ SYBR GreenER™ qPCR SuperMix Universal from Thermo Fisher Scientific in Waltham, MA. The qRT-PCR was performed on the Bio-Rad CFX96 Touch™ Real-Time PCR Detection System. The expression levels of the RNA samples were determined using the threshold cycle (ΔΔCT) method and normalized to the housekeeping gene glyceraldehyde-3-phosphate dehydrogenase (Gapdh). The primer sequences are displayed in **Supplementary Table 3**.

### RNA sequencing and bioinformatics analysis

RNA-Seq was performed in biological triplicates in IEC4.1 cells treated with siR_Ctrl or siR_Nostrill. Total RNA molecules were isolated using Trizol reagent. The BGI Americas Corporation in Cambridge, MA, carried out transcriptome sequencing and data processing using DNBSEQ™ sequencing technology platforms. To assess RNA quality, a 2100 Bioanalyzer from Agilent Technologies was employed. RNA samples meeting the criteria of consistent visual RNA patterns, an RNA Integrity Number (RIN) greater than 7, and a concentration exceeding 20 ng/μl were categorized as having good quality. The complete RNA was broken down into smaller fragments, and specific mRNA molecules were concentrated using magnetic beads with oligo (dT) sequences. Subsequently, complementary DNA (cDNA) was synthesized from the enriched mRNA. This double-stranded cDNA underwent purification and was amplified using PCR. The sequenced library products were processed on the DNBSEQ-500 platform with paired-end reads of 100 base pairs, generating an average of approximately 4.94 Gb per sample. To ensure data quality, subpar reads were excluded. This included reads with base qualities lower than 10 for over 20% of their bases, reads with adaptors, and those containing more than 5% bases marked as unknown (N bases). This filtration was achieved using internal software called SOAPnuke (version: v1.5.2) on the DNBSEQ-500 platform, with specific parameters (-I 15-q0.5-n0.1). The resultant high-quality reads were stored in the FASTQ format. Reads were aligned to the Mus_musculus genome (GCF_000001635.26_GRCm38.p6) from the NCBI database using two different software tools: HISAT2 (version: v2.0.4) and Bowtie2 (version: v2.2.5). The alignment was guided by parameters tailored to the specifics of the process (-phred64-sensitive-no-discordant-no-mixed-I 1-X 1000). Raw RNA-seq data were further refined by filtering out unreliable sites, accomplished through the application of the GATK program. This step targeted the removal of low-quality reads from each sample. Finally, the gene read counts were normalized to RPKM, which represents the expression level of genes in terms of reads per kilobase of transcript per Million mapped reads. This normalization allowed for meaningful comparison of relative gene expression levels across samples.

Differential gene-expression analyses were conducted using DESeq2. Differences in gene expression, whether increased or decreased, were quantified using logarithmically transformed fold change values. These values were represented as either log2FC (log base 2) or log10FC (log base 10), which were computed as the logarithmic difference between gene expression values under distinct treatment conditions (log2FC = log2[B] - log2[A] or log10FC = log10[B] - log10[A], where A and B denote the gene expression values). GO enrichment analysis was performed using PANTHER. To further analyze the data, Gene Set Enrichment Analysis (GSEA) was conducted using the “clusterProfiler” R package. This analysis was carried out in a pre-ranked manner, where all genes were ranked based on their log2FC values derived from the differential expression analysis. The weighted Kolmogorov-Smirnov test was applied, and p-values were adjusted using the Benjamini-Hochberg method. The findings from GSEA were depicted visually using the “ggplot2” R package, offering insights into patterns of gene set enrichment. Additionally, a volcano plot was generated with the “ggplot2” package, utilizing log2FC and adjusted p-values from the differential expression analysis to visually convey the significance and extent of gene expression alterations. For a comprehensive overview of gene expression trends, heatmaps were generated using the “ComplexHeatmap” R package. These heatmaps visually summarized gene expression patterns across different conditions. Furthermore, the expression z-score was computed based on relative gene expression levels. This score aids in standardizing and comparing gene expression values across diverse samples or conditions, facilitating more meaningful interpretation of expression patterns.

### RIP assay

The formaldehyde crosslinking RNA immunoprecipitation (RIP) assay was conducted as described previously (22, 53). To summarize, the process involved the initial cleaning of lysates using 20 μl of Magna ChIP Protein A + G Magnetic Beads (Millipore, Massachusetts) that had been pre-washed with PBS. Subsequently, the pre-cleaned lysate (250 μl) was mixed with an equivalent volume of the whole cell extract buffer (250 μl). This mixture was then combined with beads coated with an antibody to Stat1 (#9172S, Cell Signaling, Danvers, MA). The entire assembly was then rotated at 4°C overnight. Following this, the mixture underwent four rounds of washing using the whole cell extract buffer supplemented with protease and RNase inhibitors. The immunoprecipitated ribonucleoprotein (RNP) complexes and the input material were subjected to digestion in RNA PK Buffer pH 7.0 (comprising 100 mM NaCl, 10 mM TrisCl pH 7.0, 1 mM EDTA, and 0.5% SDS) in the presence of 10 μg of Proteinase K. This mixture was then incubated at 50°C for 45 minutes with continuous shaking at 400 rpm. The formaldehyde cross-links were subsequently reversed through incubation at 65°C with rotation for 4 hours. RNA was isolated from the samples using Trizol, following the protocol provided by Invitrogen. The presence of RNA was evaluated using qRT-PCR, conducted with the CFX96 Touch™ Real-Time PCR Detection System from Bio-Rad. The specific primer pairs for PCR can be found in **Supplementary Table 3**.

### ChIP assay

The detailed protocol is described elsewhere (22, 54). Briefly, cells were treated with 1% formaldehyde for 10 minutes to cross-link the proteins to the DNA, preserving the protein-DNA interactions at the time of fixation. The fixed cells were collected in 1x ice-cold PBS (Phosphate-Buffered Saline) and resuspended in an SDS (Sodium Dodecyl Sulfate) lysis buffer. The genomic DNA was then fragmented into smaller pieces through sonication resulting in fragments ranging from 200 to 500 base pairs in length. One percent of the cell extract was kept aside as input. The cell extracts containing the sheared DNA are incubated overnight at 4°C with anti-Stat1 and added protein G-agarose beads to precipitate the antibody-protein-DNA complexes. The immunoprecipitates (antibody-protein-DNA complexes bound to the beads) were washed sequentially to remove any non-specific interactions. The washing steps involve low-salt buffer, high-salt buffer, LiCl buffer, and Tris EDTA buffer. The DNA-protein complexes were eluted from the beads afterwards, separating the DNA from the proteins. The proteins in the eluted solution were then digested at 45°C for 1hr using proteinase K. To detect and quantify the DNA, qRT-PCR analysis was employed, which allowed for the measurement of the DNA content, enabling the quantification of protein binding levels at particular regions of the genome.

### ChIRP assay

Chromatin isolation by RNA purification (ChIRP) analysis was conducted as described in a previous study (22, 55). In summary, chromatin was isolated after cross-linking the cells with glutaraldehyde. A set of tiling oligonucleotide probes were designed to specifically bind to the Nostrill sequence. The sequences for these probes are available in **Supplementary Table 1**. Verification of the DNA sequences in the chromatin immunoprecipitates was performed using qRT-PCR. The same primers that cover the gene promoter regions of interest, similar to the ChIP analysis, were utlized for the ChIRP analyses. As a control, a collection of scrambled oligonucleotide probes targeting LacZ (listed in **Supplementary Table 3**) was used.

### Autophagy assay

Cell Culture and Lysate Preparation: Cells were plated in six-well plates with 2 ml of medium per well to achieve 80%–90% confluency within 48h. After 24h, the culture medium was replaced with treatment (such as, IFN-γ at a dose of 10 ng/ml) or control medium and incubated for an additional 24h. Two hours prior to collection, 1:100 dilutions of the stock solutions of leupeptin (10 mM) and NH4Cl (2 M) were added to the medium to yield final concentrations of 100 μM and 20 mM, respectively. The cell pellet was collected using EDTA/PBS, washed with ice-cold PBS, and lysed with pre-cooled lysis buffer. Lysates were sonicated for 10–15 sec or treated with syringe to complete cell lysis and shear DNA to reduce sample viscosity. The lysate was centrifuged at 12,000 rpm for 10 minutes at 4°C, and the supernatant was transferred to a new tube. Protein concentration was determined, samples were adjusted for equal concentrations, mixed with loading buffer, and boiled before storing at -80°C.

For SDS-PAGE and Western blotting, equal protein amounts (10–20 μg) were loaded onto the SDS-PAGE gel with a molecular weight marker. The gel was run at 95 V for 20 minutes and then at 150 V for approximately 1–2 hours until the dye reached 1/4 inch from the bottom of the gel. LC3-II migrated at ~14 kD. The separated proteins were transferred onto a PVDF membrane, chosen for its enhanced LC3-II binding. Following transfer, the membrane was blocked with 5% BSA blocking buffer for one hour at room temperature and probed with primary anti-LC3II antibody (1:1000, cat#2775S from Cell Signaling) overnight at 4°C. The membrane was washed three times for 15 minutes each with TBST at room temperature, probed with secondary HRP-conjugated anti-rabbit IgG antibody (1:5000) for one hour at room temperature, and washed again. An ECL solution mixture was applied to the membrane, and the emitted light was captured. The levels of LC3-II in each sample were normalized to β-actin (1:1000, cat#A1978 from Cell Signaling) for analysis.

### Statistical analysis

Data are given as mean ± SEM. from at least three independent experiments or biological replicates. The two-tailed unpaired Student’s *t* test was used for compassion between two groups and one-way ANOVA were used for compassion among three or more groups. *p* values < 0.05 were considered statistically significant.

## Data availability statement

The datasets presented in this study can be found in online repositories. The names of the repository/repositories and accession number(s) can be found below: GSE245211 (GEO).

## Ethics statement

The animal study was approved by IACUC, Rush University Medical Center. The study was conducted in accordance with the local legislation and institutional requirements.

## Author contributions

ZS: Conceptualization, Data curation, Formal Analysis, Investigation, Methodology, Resources, Software, Validation, Visualization, Writing – original draft. KJ: Data curation, Investigation, Validation, Writing – review & editing. A-YG: Data curation, Formal Analysis, Investigation, Methodology, Resources, Software, Supervision, Validation, Visualization, Writing – review & editing. SD: Methodology, Software, Writing – review & editing. CP: Methodology, Visualization, Writing – review & editing. MG: Methodology, Visualization, Writing – review & editing. SW: Methodology, Visualization, Writing – review & editing. NM: Formal Analysis, Investigation, Methodology, Writing – review & editing. AS: Formal Analysis, Resources, Writing – review & editing. X-MC: Conceptualization, Data curation, Formal Analysis, Funding acquisition, Methodology, Project administration, Resources, Supervision, Validation, Writing – original draft, Writing – review & editing.

## Supplementary material

Supplementary Table 1 List of genes from RNA-Seq in IEC4.1 cells following *Cryptosporidium* infection.

Supplementary Table 2 List of genes from RNA-Seq in IEC4.1 cells following IFN-γ stimulation.

Supplementary Table 3 Primers for PCR, RIP, ChIP, ChIRP and construct generating.
