# Supplementary figures and images for: LncRNA Nostrill promotes interferon-γ-stimulated gene transcription and facilitates intestinal epithelial cell-intrinsic anti-Cryptosporidium defense
Source: Front Immunol. 2024 Jul 8;15:1397117. doi: 10.3389/fimmu.2024.1397117 (PMC11260782; doi:10.3389/fimmu.2024.1397117)

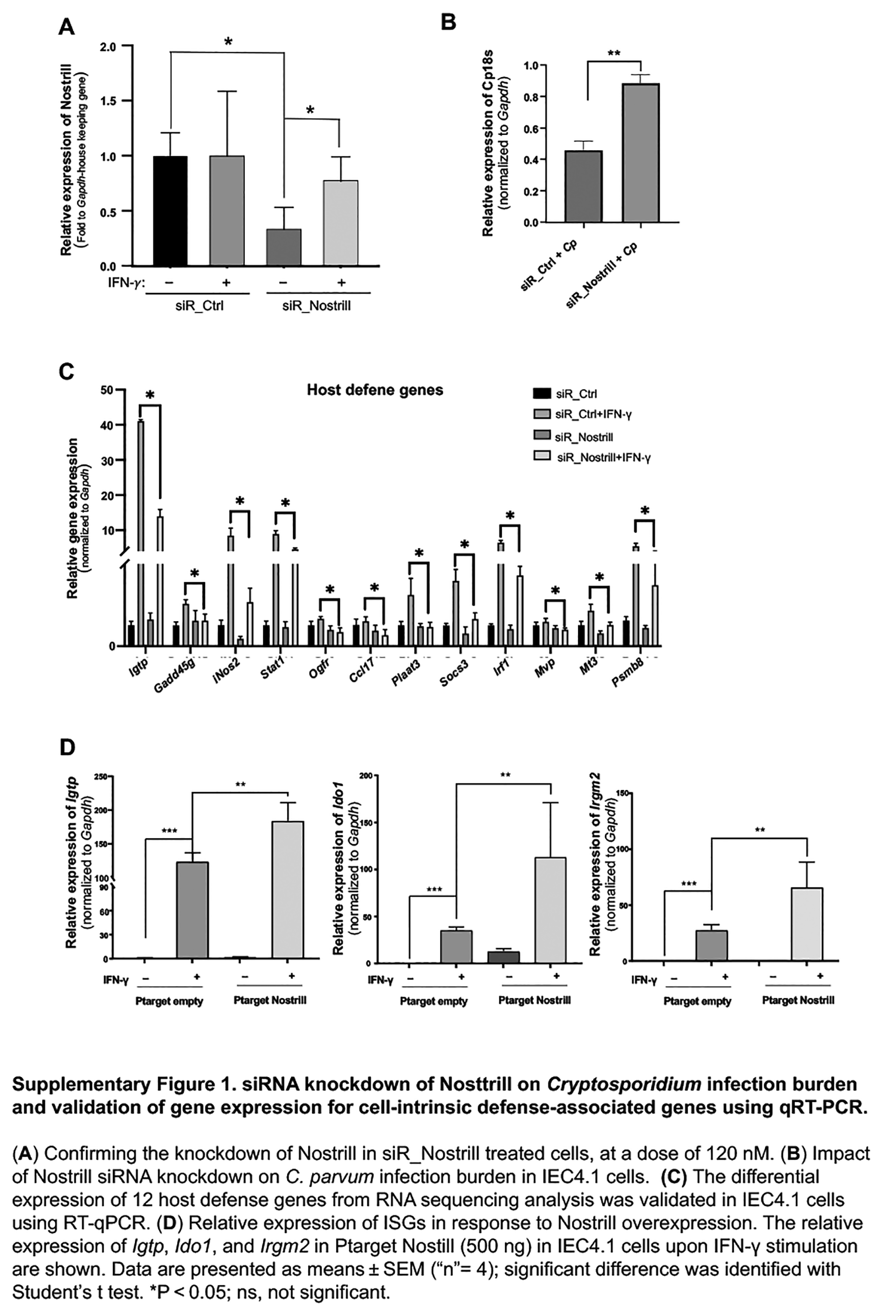

Supplement: Supplementary file 1 [file Image_1.tif]

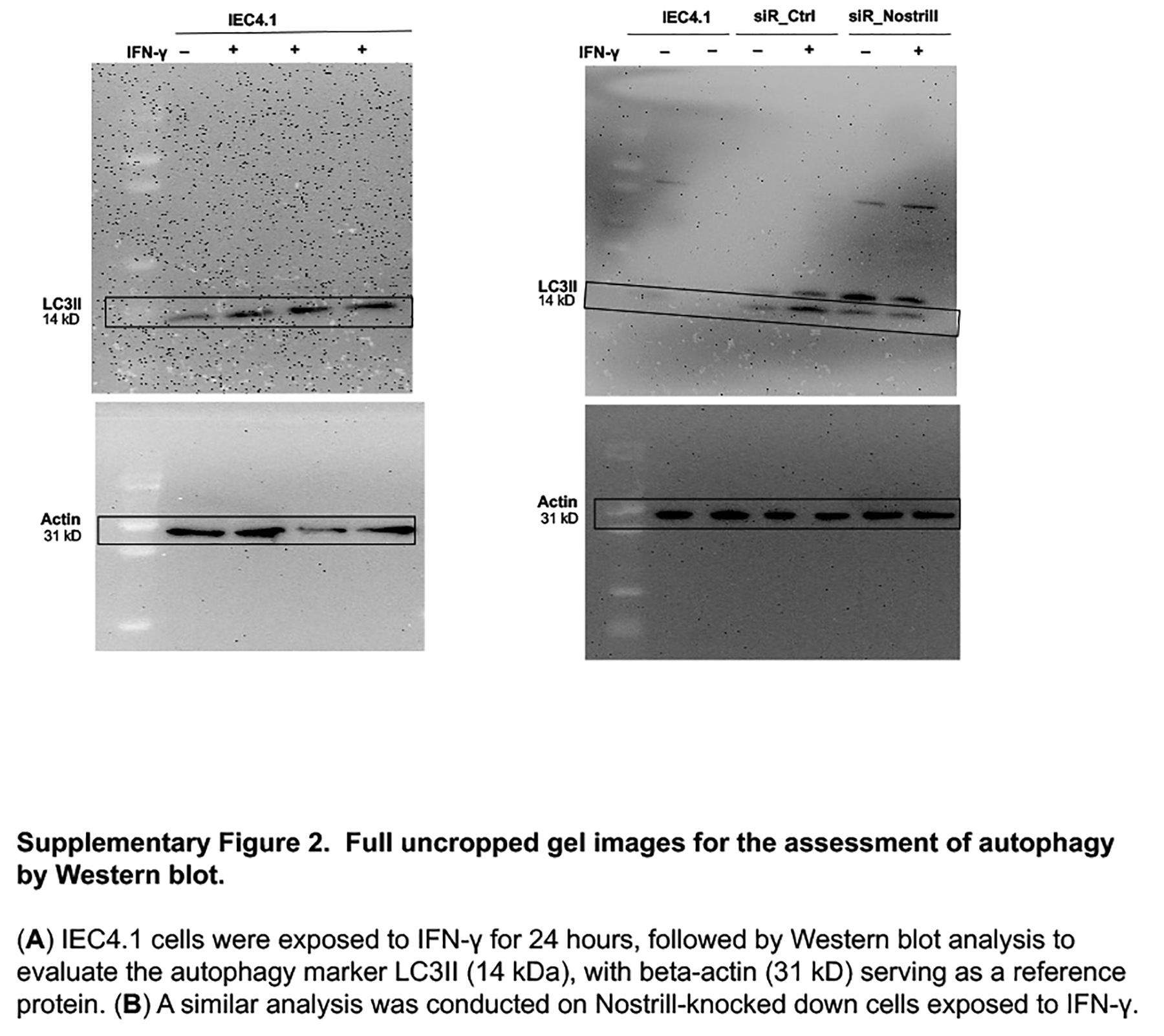

Supplement: Supplementary file 2 [file Image_2.tif]

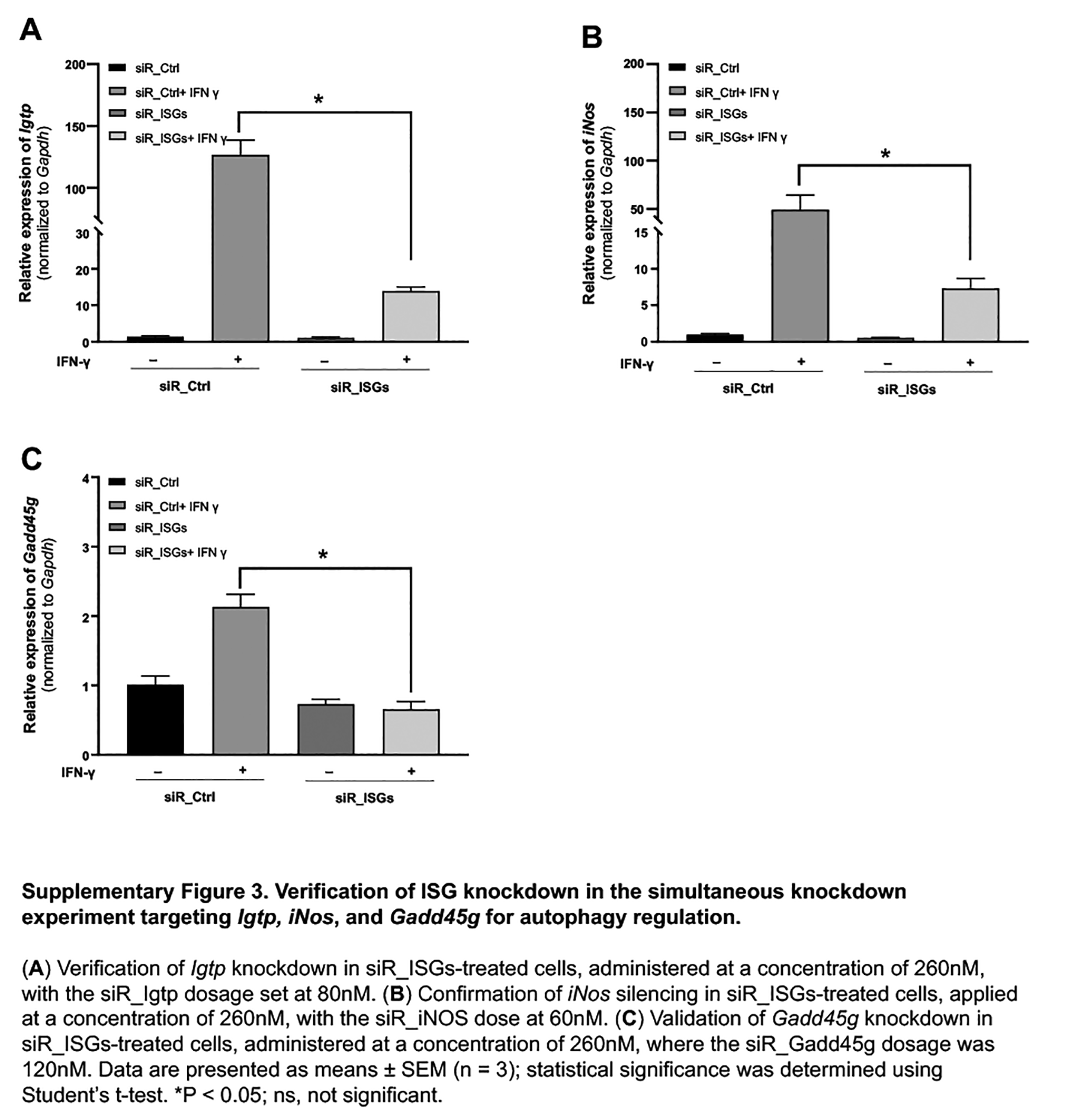

Supplement: Supplementary file 3 [file Image_3.tif]
